# Effectiveness of Magnifying Narrow-Band Imaging Endoscopy for Differential Diagnosis between the High-Risk Mixed-Type and Low-Risk Simple-Type of Low-Grade, Well-Differentiated Gastric Tubular Adenocarcinoma

**DOI:** 10.1155/2016/3028456

**Published:** 2016-04-14

**Authors:** Takashi Saitoh, Asako Takamura, Gen Watanabe, Suzuko Sugitani, Yoichi Ajioka

**Affiliations:** ^1^Department of Gastrointestinal Endoscopy and Gastroenterology, Niigata Prefectural Federation of Japan Agricultural Cooperatives, Toyosaka Hospital, 1-11-1 Isurugi, Kita-ku, Niigata 950-3327, Japan; ^2^Division of Gastrointestinal Endoscopy and Gastroenterology, Niigata Prefectural Kamo Hospital, 1-9-1 Aomi-cho, Kamo, Niigata 959-1326, Japan; ^3^Division of Molecular and Diagnostic Pathology, Niigata University Graduate School of Medical and Dental Sciences, 1-757 Asahimachi-dori, Chuo-ku, Niigata 951-8510, Japan

## Abstract

*Backgrounds*. Magnifying endoscopy with narrow-band imaging (NBI-ME) is useful for diagnosing differentiated early gastric cancer (D-EGC). D-EGC is classified as high- or low-grade based on its glandular architectural and cytological atypia. Low-grade, well-differentiated tubular adenocarcinoma (LG-tub1) mixed with high-grade tub1 (HG-tub1) and/or other histological types (M-LG-tub1) may indicate a primitive high-risk malignant lesion compared to histologically simple-type LG-tub1 (S-LG-tub1). Because LG-tub1 is occasionally difficult to diagnose due to its unclear demarcation under conventional white light endoscopy, early precise diagnoses are important.* Methods*. We compared NBI-ME and postendoscopic submucosal dissection histological findings for 30 S-LG-tub1 and 15 M-LG-tub1 lesions. We classified the NBI-ME findings of S-LG-tub1 (and not D-EGC) into four patterns. The differential diagnosis between M-LG-tub1 and S-LG-tub1 depended on the presence of more than one of these patterns without or with other patterns (referred to as “limited-to-four-pattern [LFP] sign-positive” and “sign-negative”, resp.).* Result*. The sensitivity, specificity, accuracy, positive and negative predictive values, and intraobserver and interobserver agreement, using the “LFP sign” for the differential diagnosis between M-LG-tub1 and S-LG-tub1, were 87.9%, 91.7%, 88.9%, 96.7%, 73.3%, and* k* = 0.842 and* k* = 0.737, respectively.* Conclusion*. NBI-ME may be useful in differentiating between high-risk M-LG-tub1 and low-risk S-LG-tub1.

## 1. Introduction

Magnifying endoscopy with narrow-band imaging (NBI-ME), which has recently entered widespread use, is useful for diagnosing the lateral spread and histological type of early gastric cancer (EGC) [1–7].

The potential for submucosal (SM [8]) invasion and lateral spread in differentiated early gastric cancer [9] (D-EGC) is an important consideration for this malignancy, as SM invasion is associated with the risk of lymph node metastasis and lateral spread is associated with the risk of recurrence and residual disease after endoscopic and surgical resection [10, 11]. D-EGC is subclassified into histological types based on the structural atypia grade (papillary adenocarcinoma [pap], well-differentiated tubular adenocarcinoma [tub1], or moderately differentiated tubular adenocarcinoma [tub2] [8]). D-EGC is also classified as low- or high-grade cancer based on its glandular architectural and cytological atypia grade [12, 13]. Some low-grade (LG-) tub1 (LG-tub1) lesions can transform into malignant lesions with SM invasion and lateral spread [14–17]. It is also well known that other histological types and atypia grades of gastric cancer have higher potential for SM invasion and lateral spread than LG-tub1 [9, 12] and that the larger in size D-EGC lesion with gastric mucin phenotype becomes the more undifferentiated types of cancer components with gastric mucin phenotype increase [18, 19]. LG-tub1 occasionally appears to coexist with high-grade tub1 (HG-tub1) and/or with other histological types. Thus, LG-tub1 mixed with HG-tub1 and/or with other histological types (M-LG-tub1) can be considered a primitive high-risk malignant lesion with the potential for SM invasion and lateral spread. Early precise diagnosis and careful treatment are important because LG-tub1 (composed of simple-type [S-] and mixed-type [M-] LG-tub1) is reported to present occasionally with an unclear border on white light endoscopy (WLE) and in resected specimens [12, 20–22].

Incidentally, NBI-ME pattern types reflect histological types, microvessels running along the intervening part of gastric carcinomatous epithelia, and possibly glandular architectural and cytological atypia grades. Therefore, the NBI-ME pattern types of S-LG-tub1 can be considered the simplest because S-LG-tub1 exhibits the lowest structural, glandular architectural, and cytological atypia grades and variety of microvascular patterns among gastric cancers. The higher structural, glandular architectural, and cytological atypia grade a D-EGC lesion has, the greater the variations of NBI-ME types of S-LG-tub1 that may occur in that lesion. Accordingly, glandular architectural and cytological atypia grades may have to be considered as well as histological types and subtypes and microvascular patterns when classifying NBI-ME findings for the diagnosis of D-EGC.

Based on the above views and previous reports [1–7, 22, 23], we conducted a first study to examine NBI-ME findings for LG-tub1.

## 2. Patients and Methods

### 2.1. Acronyms

Many acronyms are used in this report to avoid redundant sentences and shorten the paper; the full list of acronyms is shown in Table 1.

### 2.2. Subjects and Ethics Statement

**Figure 1:**
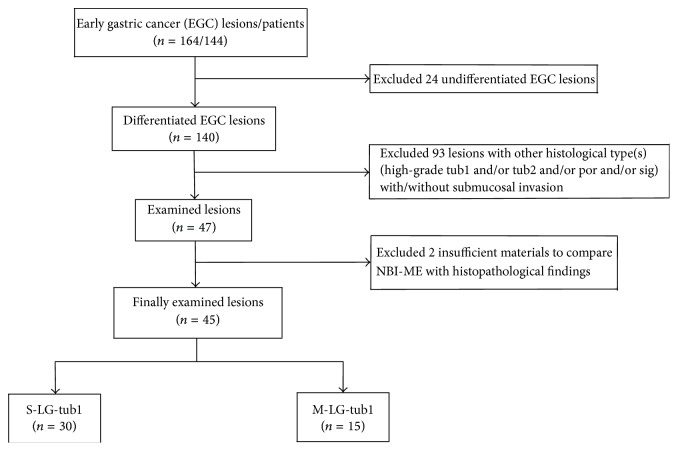
The investigation flow. Firstly, twenty-four undifferentiated early gastric cancer (EGC) lesions were excluded. Secondly, ninety-three lesions were excluded because they were composed of different histological types from low-grade- (LG-), well-differentiated tubular adenocarcinoma (tub1) (LG-tub1) with or without submucosal (SM) invasion. Thirdly, two lesions were excluded because of heat-induced degeneration during endoscopic submucosal dissection (ESD) and hence an insufficient amount of material for making comparisons. Finally, we were able to perform detailed comparisons of magnifying endoscopy with narrow-band imaging (NBI-ME) and post-ESD histological findings for 30 simple-type- (S-) LG-tub1 (S-LG-tub1) and 15 mixed-type- (M-) LG-tub1 (M-LG-tub1) lesions.

Of 11847 patients who underwent esophagogastroduodenoscopies in our department between September 2008 and September 2015, we examined 164 lesions (140 differentiated and 24 undifferentiated lesions [9]) from 144 patients. We synthetically diagnosed the examined lesions as superficial EGCs [8] by WLE plus chromoendoscopy and NBI-ME findings and referred to the pathological findings of the resected specimens as a gold standard. Finally, we were able to perform detailed comparisons of NBI-ME and post-ESD histological findings for 30 S-LG-tub1 and 15 M-LG-tub1 lesions (Figure 1). Written informed consent was obtained from each patient before the first endoscopic examination and therapy. This study was approved by the ethics committee at our hospital.

### 2.3. Patient Characteristics

The clinicopathological features of the subjects, additionally including grade of background mucosal atrophy (Kimura-Takemoto classification [24]), NBI-ME pattern types, ability to recognize the lateral border on WLE and NBI-ME, mucin phenotype, and presence or absence of* H. pylori* infection, were examined. The lateral border was considered unclear if any part was not apparent, even slightly, on WLE and NBI-ME. As eradication was achieved in all of the* Helicobacter pylori-* (*H. pylori-*) positive cases after NBI-ME observation and ESD, eradication did not affect the NBI-ME findings of such cases in both the S- and M-LG-tub1 groups [25].

### 2.4. Endoscopy

Endoscopies were performed by a single endoscopist who had 26 years of endoscopic experience and was skillful in WLE plus chromoendoscopy and NBI-ME using a GIF-H260Z magnifying upper gastrointestinal endoscope (Olympus Medical Systems, Tokyo, Japan) and two electronic endoscopy systems (EVIS LUCERA Spectrum and EVIS LUCERA ELITE Spectrum; Olympus Medical Systems). When NBI-ME was performed, a soft black hood (MB-46; Olympus Medical Systems) was mounted on the tip of the endoscope [26].

### 2.5. Endoscopic Submucosal Dissection (ESD)

ESDs were performed by the same endoscopist. The GIF-260Z system was also used for ESD. To enable detailed comparisons with postremoval histopathological findings, marks were made around the lesions, and images were captured before and after with WLE and NBI-ME.

### 2.6. NBI-ME Findings

#### 2.6.1. Definitions of the Four NBI-ME Pattern Types

The highly magnified views and the characteristic microvascular and microstructural findings of the four NBI-ME pattern types are shown in Table 2.

- Regular mesh pattern (RMP) is characterized by tightly connected microvessels that form regular fine-networked patterns (Table 2(a)).
- Loops within irregular polygonal and papillary structures (LIPPS) are typically characterized by loop-shaped microvessels within irregular polygonal and papillary shaped mucosal structures surrounded by bold white lines. Each section of LIPPS surrounded by bold white lines is located close to other sections (Table 2(b)).
- Maze-like pattern (MLP) appears as a partition of microvessels that divide the path of the bold white line, which is slightly bolder than that observed in LIPPS. The two-dot line demarcates the carcinomatous and noncarcinomatous areas (Table 2(c)).
- Ultra-fine granular pattern (u-FGP) is characterized by the presence of surface structures composed of ultra-fine granules with slightly uneven sizes and distributions and microvessels that are perceived as minute brownish points [22, 23] (Table 2(d)).

#### 2.6.2. Definitions of NBI-ME Pattern Variations

**Figure 2:**
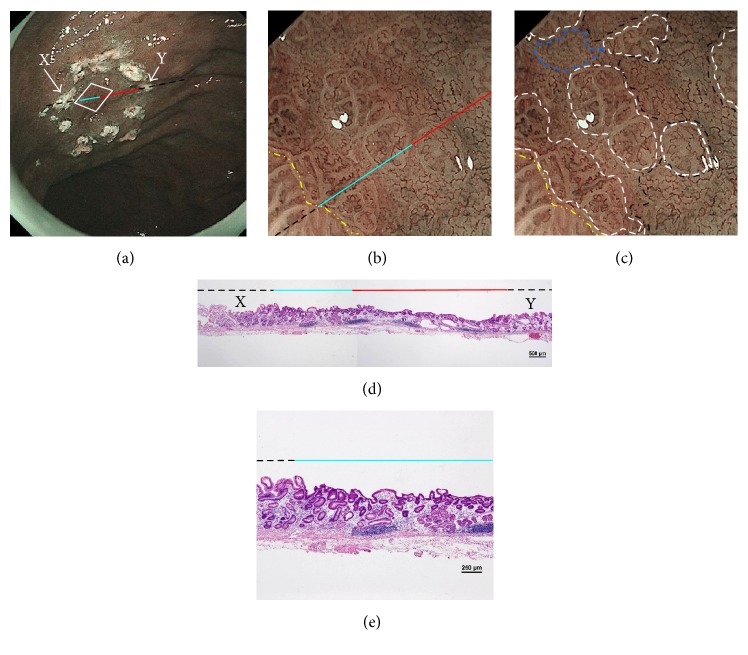
Endoscopic and histopathological findings of simple-type- (S-), low-grade- (LG-), well-differentiated tubular adenocarcinoma (tub1) (S-LG-tub1). (a) Low-magnification view of narrow-band imaging magnifying endoscopy (NBI-ME) shows a superficial depressed (0-IIc) lesion. The thin-broken, thin-solid, and thick-solid lines show the slice line within the specimen resected by endoscopic submucosal dissection (ESD). The thin-broken, thin-solid, and thick-solid lines indicate the noncarcinomatous part, the same carcinomatous part that is shown in (b), (d), and (e), and the same carcinomatous part that is shown in (b) and (d), respectively. The slice line was decided along with those lines which linked X to Y cautery markings. The X and Y markings are shown in (a) and (d) as landmarks in the NBI view and HE-stained histological specimens, respectively. (b) High-magnification NBI-ME (boxed area in (a)). The two-dot chain line demarcates the carcinomatous and noncarcinomatous areas. The thin-broken, thin-solid, and thick-solid lines are the same lines in (a), (d), and (e). (c) The same view of (b) reveals regular mesh pattern (RMP) with loose mesh pattern (LMP), loops within irregular polygonal and papillary structures (LIPPS), ultra-fine granular pattern (u-FGP) (surrounded by one-dot chain, short-dotted, and broken lines, resp.), and a “minute transitional zone” (outside the lines) in a single lesion. The two-dot chain line demarcates the carcinomatous and noncarcinomatous areas. The limited-to-four-pattern (LFP) sign-positive lesions included a “minute transitional zone” between any of the four aforementioned types, whereas the LFP sign-negative lesions apparently included other patterns that could not be classified into any of the four aforementioned types. The width of a “minute transitional zone” was defined as <500 *μ*m; because the highest-magnification view is 4 × 4 mm, we could recognize the widths of a “minute transitional zone” less than one-eighth of the quadrangular side, which is 500 *μ*m. This lesion was diagnosed by NBI-ME as a simple-type- (S-), low-grade- (LG-), well-differentiated tubular adenocarcinoma (tub1) (S-LG-tub1). (d) Low-magnification view of a post-ESD specimen and (e) moderately magnified view of a post-ESD specimen present S-LG-tub1. Histological examination corroborated the NBI-ME diagnosis.

Loose mesh pattern is characterized by loosely connected microvessels that form loose-networked mesh patterns, observed in both S- and M-LG-tub1, and is considered to be a variation of RMP. The running of microvessels somewhat resembles an untangled thread in some parts (Figure 2(b)).

Loop-shaped microvessels within irregular long-oval- and tubular-shaped mucosal structures surrounded by bold white lines as variations of LIPPS are occasionally shown in S-LG-tub1 lesions (not shown).

#### 2.6.3. Definition of NBI-ME Additional Factor

Visible orifice of carcinomatous crypt (VOCC) is a small, round, or oval structure that occurs in the center of each microvascular network (Table 2(a)). We previously reported that VOCC is visible on NBI-ME without sprinkling acetic acid in vivo when its width is between 30 and 70 *μ*m for the first time [22].

### 2.7. Examination Procedure of the Four NBI-ME Patterns

**Figure 3:**
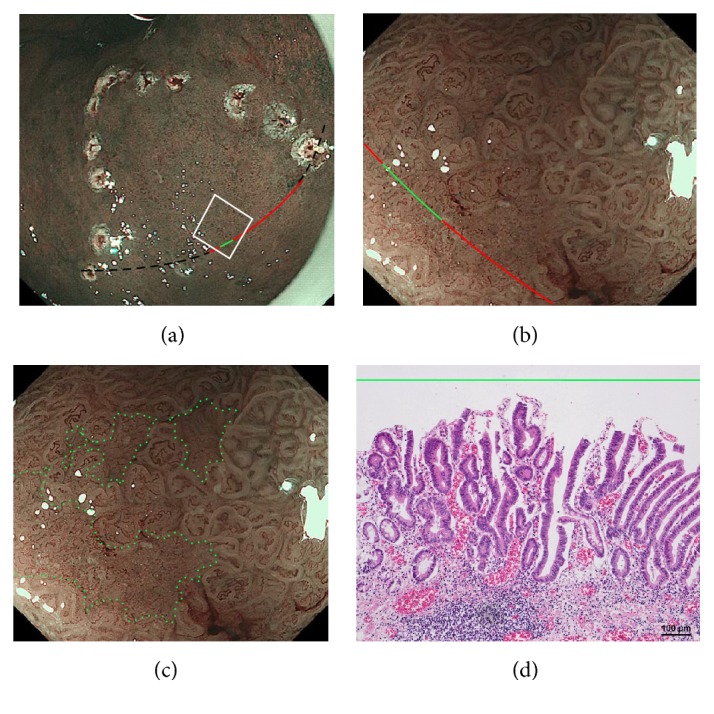
Endoscopic and histopathological findings of mixed-type- (M-), low-grade- (LG-), well-differentiated tubular adenocarcinoma (tub1) (M-LG-tub1). (a) Low-magnification view of narrow-band imaging magnifying endoscopy (NBI-ME) shows a superficial flat (0-IIb) lesion surrounded by cautery markings. The thin-broken, thin-solid, and thick-solid gentle curves show the one slice within the specimen resected by endoscopic submucosal dissection (ESD). The thin-broken, thin-solid, and thick-solid gentle curves indicate the noncarcinomatous part, the same carcinomatous part that is shown in (b), and the same carcinomatous part that is shown in (b) and (d), respectively. The slice was decided along with those gentle curves that changed into a single straight line linking two cautery markings because of being stretched out in the removed specimens. (b) High-magnification NBI-ME (boxed area in (a)). The thin-solid and thick-solid gentle curves show the same curves in (a) and (d). (c) The same view of (b) reveals another pattern that could not be classified into any of the four NBI-ME patterns (regular mesh pattern (RMP), loops within irregular polygonal and papillary structures (LIPPS), maze-like pattern (MLP), and ultra-fine granular pattern (u-FGP)) and LIPPS (surrounded by small-dotted lines and outside the lines, resp.) in a single lesion. This lesion had LIPPS, the aforementioned pattern, and RMP with loose mesh pattern (LMP) in different parts from this view and was diagnosed as mixed-type- (M-), low-grade- (LG-), well-differentiated tubular adenocarcinoma (tub1) (M-LG-tub1) (Figure 3(d)). (d) A post-ESD specimen presents a mixed-type- (M-), low-grade- (LG-), well-differentiated adenocarcinoma (tub1) (M-LG-tub1); the same part of the thick-solid gentle curve in (b) (within the area surrounded by small-dotted lines in (c)) is shown and the specimen exhibits a mixed lesion of low-grade, well-differentiated tubular adenocarcinoma (LG-tub1) with high-grade, well-differentiated tubular adenocarcinoma (HG-tub1) (Figure 3(d)). Histological examination corroborated the NBI-ME diagnosis.

The NBI-ME findings of all examined lesions were retrospectively examined by two highly experienced endoscopists. The same two endoscopists who were blinded to the prior histopathological diagnosis of S- and M-LG-tub1 reviewed the NBI-ME images. For each lesion, the observers classified the NBI-ME findings as exhibiting more than one of the four aforementioned NBI-ME patterns without or with other patterns that could not be classified into any of the four aforementioned types (termed “limited-to-four-pattern [LFP] sign-positive” and “LFP sign-negative,” resp.). The LFP sign-positive and sign-negative lesions were diagnosed as S-LG-tub1 (Figures 2(a)–2(d)) and M-LG-tub1 (Figures 3(a)–3(d)), respectively.

Following these evaluations, the two endoscopists conducted a consensus review of discrepant lesions to reach a consensus diagnosis, if possible. The differential diagnosis between S- and M-LG-tub1 depended on the presence of LFP sign. At least 2 months after the first review, one observer reviewed the NBI-ME findings again. The accuracy, sensitivity, specificity, positive predictive value (PPV), and negative predictive value (NPV) were calculated. The interobserver agreement of NBI-ME using the four aforementioned types for the diagnosing of S-LG-tub1 was assessed. The intraobserver and interobserver agreements of NBI-ME using the LFP sign diagnostic system for the differential diagnosis between S- and M-LG-tub1 were also assessed.

### 2.8. Histopathological Specimens

Histopathological sections were prepared for comparison with the post-ESD specimens. Formalin (10%) was used to fix the 45 LG-tub1 lesions that were obtained by ESD. Following paraffin embedding, thin sections were prepared, and hematoxylin and eosin (HE) staining and immunostaining (CD10, mucin 2 [MUC2], MUC6, MUC5AC, caudal type homeobox 2 [CDX2], Ki-67, and p53) were performed. The following antibodies were used: CD10, MUC2, MUC6, MUC5AC, p53 (Novocastra, Newcastle, UK), CDX2, and Ki-67 (Dako Japan, Tokyo, Japan). Two highly experienced pathologists made histopathological diagnoses based on HE-stained specimens. Atypia grades were assigned as previously described [12]. Mucin phenotypes were determined as previously reported [27, 28]. Staining for carcinoma cell proliferative capacity (Ki-67) and a tumor suppressor gene (p53) served as references for diagnosing the atypia grade [21].

### 2.9. Statistical Analysis

SPSS version 22.0 (IBM Japan, Tokyo, Japan) was used for the statistical analysis. The *t*-test was used to compare age and size. The Chi-squared test was used to compare sex, macroscopic type, location, and colors on WLE among groups, grade of background mucosal atrophy, and mucin phenotype and* H. pylori* status of the LG-tub1 types. McNemar's test was used to compare the ability to discern the lateral extent of the lesions on WLE compared to NBI-ME. A *p* value of <0.05 was considered statistically significant. The diagnostic accuracy, sensitivity, specificity, PPV, and NPV were calculated for each observer. The intraobserver and interobserver variability were calculated using Cohen's kappa statistic for the NBI-ME pattern diagnosis of S-LG-tub1 and the differential diagnosis between S-LG-tub1 and M-LG-tub1 via NBI-ME.

## 3. Results

### 3.1. Patient Characteristics

The clinical characteristics and WLE findings are shown in Table 3. There were no significant differences between S- and M-LG-tub1 in age, sex, macroscopic type, location, color on WLE, endoscopic atrophic pattern, and* H. pylori* status. Mean size of lesions in the M-LG-tub1 group was significantly higher than that of lesions in the S-LG-tub1 group (*p* = 0.038). Lateral border was more clearly recognizable on NBI-ME (93.3%) than WLE (73.3%) in the S-LG-tub1 group (*p* = 0.004). Active* H. pylori* infection was detected in 53.8% of the patients. After NBI-ME observation and ESD,* H. pylori* was successfully eradicated in all positive patients.

### 3.2. ESD

Complete curative ESD was confirmed for all 45 of the resected lesions via histological examination. No patient exhibited either lymphatic permeation or SM invasion.

### 3.3. NBI-ME Findings

To comprehensively describe the NBI-ME findings for S-LG-tub1, RMP, LIPPS, MLP, and u-FGP were defined. In the S-LG-tub1 group (30 lesions), RMP, LIPPS, MLP, and u-FGP accounted for 66.7%, 10.0%, 13.3%, and 10.0% of the lesions, respectively (Table 3).

Consequently, the interobserver agreement of NBI-ME using the four pattern types for the diagnosis of S-LG-tub1 was high (*p* = 0.859) (Table 4). The sensitivity, specificity, accuracy, PPV, and NPV, as well as intraobserver and interobserver agreement, using the LFP sign for the differential diagnosis between M- and S-LG-tub1 were 87.9%, 91.7%, 88.9%, 96.7%, 73.3%, and* k* = 0.842 and* k* = 0.737, respectively (Table 5).

### 3.4. Histopathology

Among the 45 HE-stained lesion specimens, 15 were diagnosed as M-LG-tub1 and 30 as S-LG-tub1. Immunohistochemistry revealed that the frequency of lesions of the gastric (G) phenotype in the M-LG-tub1 group (53.3%) and the intestinal (I) phenotype in the S-LG-tub1 group (56.7%) were significantly higher and the gastric (G) phenotype in the S-LG-tub1 group (6.7%) was significantly lower than that of other phenotypes in both groups (*p* = 0.007) (Table 3).

Although a NBI-ME finding that may be specific to pap was also reported [6], it revealed that no component of pap was included among the histologically examined specimens after ESD.

## 4. Discussion

This report is the first to compare differences in NBI-ME findings among lesions with similar histologies and atypia grades (LG-tub1).

Prior reports indicated the possibility that the histological type of gastric cancer is reflected in the NBI-ME findings [2–6, 22, 23]. Nakayoshi et al. classified NBI-ME patterns of depressed-type EGC lesions as the fine network pattern (FNP) and the corkscrew pattern (CSP) (also referred to as D-EGC and undifferentiated type gastric cancer, resp. [2, 9]). NBI-ME findings of D-EGC as the “mesh pattern” and “loop pattern” for diagnosing D-EGC were previously reported [3]. NBI-ME findings of D-EGC are considered to differ among histological subtypes. Moreover, atypia grades of D-EGC may be reflected in the NBI-ME findings because the backscattering of the NBI beam from carcinomatous epithelial cells (cell walls, cytoplasms, and nuclei) and intercellular substances and the absorption of the NBI beam into microvessels running along the intervening part of carcinomatous epithelia may be considered to differ among atypia grades.

It can be difficult to represent NBI-ME findings for D-EGC as a single pattern type [2–5, 22]. It is well known that there are sometimes different histological subtypes and/or types within a single D-EGC lesion (i.e., tub1 with tub2 and tub1 with tub2 and partly with undifferentiated cancer components). Therefore, if there are several histological types of cancer mixed within a single D-EGC lesion, the NBI-ME findings in the single D-EGC lesion will exhibit variations and combinations of several NBI-ME pattern types according to the predominant histological types of cancer at different observation points. Moreover, under the hypothesis that different atypia grades yield different NBI-ME findings, there could be variations and combinations of several types of the aforementioned four NBI-ME patterns, even in a single LG-tub1 lesion mixed with HG-tub1 components. It may be necessary to consider atypia grades when classifying NBI-ME findings for diagnosing D-EGC because high-grade cancer has more malignant potential than low-grade cancer. Therefore, it may be necessary to determine the diagnostic NBI-ME patterns based on each of different histological subtypes, and types and glandular architectural and cytological atypia grades.

In this study, we distinguished between tub1 and tub2 among D-EGC, additionally between LG- and HG-tub1 among tub1, and even between M- and S-LG-tub1 among LG-tub1. There appear to be some D-EGC lesions with NBI-ME findings in previous reports that are not compatible with the patterns described in this report. Some of the D-EGC lesions that were previously reported may be variations of the aforementioned four NBI-ME patterns of S-LG-tub1.

We proposed RMP, LIPPS, MLP, and u-FGP classifications for the diagnosis of S-LG-tub1 for the first time. We limited the histological type and glandular architectural and cytological atypia grade of our 30 specimens to S-LG-tub1, which may have allowed us to demonstrate RMP, LIPPS, MLP, and u-FGP as more “pure” NBI-ME findings in LG-tub1 than in the other histological subtype and glandular architectural and cytological atypia grade of D-EGC and in undifferentiated gastric cancer. Under the hypothesis that the NBI-ME pattern types of S-LG-tub1 could be represented by these four pattern types, it is possible that S-LG-tub1 is LFP sign-positive and M-LG-tub1 is LFP sign negative. Additionally, NBI-ME was found to be a more accurate diagnostic tool for determining the lateral extent of LG-tub1 than WLE. Therefore, this study is clinically important, as it aims to differentially diagnose between the high-risk M-LG-tub1 and low-risk S-LG-tub1 and accurately diagnose lateral extent, which enables early detection and precise treatment before lymphatic permeation and SM invasion and caution to be exercised for high-risk M-LG-tub1 before and after treatment. It is also important in terms of basic science, as it involves translational research that enables comparisons between NBI-ME and pathological findings.

The frequency of lesions of the G phenotype was significantly higher in the M-LG-tub1 (53.3%) and lower in the S-LG-tub1 group (6.7%) (*p* = 0.007). In other words, this suggests that M-LG-tub1 lesions could pose a higher risk, in terms of malignant potential, than S-LG-tub1 lesions among LG-tub1.

From the perspective of early detection and treatment, it is important to differentiate between M- and S-LG-tub1 at diagnosis. More LG-tub1 lesions must be investigated in order to establish the effectiveness of NBI-ME for the differential diagnosis between M- and S-LG-tub1.

## 5. Conclusions

In this study, the histological type and atypia level were limited to LG-tub1, and the NBI-ME findings of S-LG-tub1 were classified as RMP, LIPPS, MLP, and u-FGP. The LFP sign on NBI-ME may be useful with high sensitivity, specificity, accuracy, PPV, NPV, and intraobserver and interobserver agreement for the differential diagnosis in the intramucosal early stage between the high-risk M-LG-tub1 and low-risk S-LG-tub1, the former of which has the higher frequency of the G mucin phenotype and is considered to have higher malignant potential. Accordingly, accurate differential diagnosis between high-risk M-LG-tub1 and low-risk S-LG-tub1 via NBI-ME may lead to more careful follow-up for lymph node metastasis after appropriate endoscopic treatment for intramucosal gastric cancer and our diagnostic system by NBI-ME may contribute to a qualitative diagnosis of some D-EGCs.

## Figures and Tables

**Table 1:** List of acronyms by category.

Acronyms	Full spelling
(1) Endoscopy	
ESD	Endoscopic submucosal dissection
NBI-ME	Magnifying endoscopy with narrow-band imaging
WLE	White light endoscopy
(2) NBI-ME findings	
LFP sign	Limited-to-four-pattern sign
LIPPS	Loops within irregular polygonal and papillary structures
MLP	Maze-like pattern
RMP	Regular mesh pattern
u-FGP	Ultra-fine granular pattern
VOCC	Visible orifice of carcinomas crypt
(3) Histopathology	
EGC	Early gastric cancer
D-EGC	Differentiated early gastric cancer
HG-tub1	High-grade, well-differentiated tubular adenocarcinoma
LG-tub1	Low-grade, tub1
M-LG-tub1	Mixed-type, LG-tub1
S-LG-tub1	Simple-type, LG-tub1
(4) Statistics	
PPV	Positive predictive value
NPV	Negative predictive value

Many acronyms are used in this report to avoid redundant sentences and shorten the paper; the full list of acronyms is shown in Table 1.

**Table 2:** Summary of the microvascular and microstructural items and high-magnification views of the four magnifying endoscopy with narrow-band imaging (NBI-ME) pattern types are shown.

NBI-ME classification	Microvascular				Microstructural	
	Pattern	Diameter	Running	Disruption	Pattern	VOCC
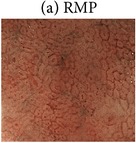	Regular mesh	Irregular	Symmetric	Absent	Imperceptible	Perceptible (or imperceptible)

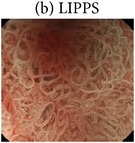	Loops	Irregular	Asymmetric	Absent	Irregular polygonal and papillary	None

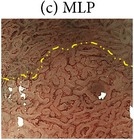	Maze-like	Irregular	Asymmetric	Absent	Maze-like	Partly perceptible (or imperceptible)

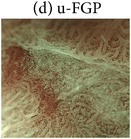	Very small dots	Imperceptible	None	Absent	Ultra-fine granular	None

(a) Regular mesh pattern (RMP). The microvessels are tightly connected and form regular mesh. Of the microvessels, the diameters are irregular and the running is symmetric. Microstructural pattern is imperceptible. Visible orifice of carcinomatous crypt (VOCC) is perceptible in this lesion. We previously reported for the first time that VOCC is visible on NBI-ME without sprinkling acetic acid in vivo when its width is between 30 and 70 *μ*m [22].

(b) Loops within irregular polygonal and papillary structures (LIPPS). Typical LIPPS are presented. The microvessels in LIPPS form loops. Of the microvessels, the diameters are irregular and the running is asymmetric. Each section of LIPPS surrounded by bold white lines is located close to other sections.

(c) Maze-like pattern (MLP) characterized by a partition of microvessels that divide the path of the bold white line, which is slightly bolder than the line observed in LIPPS. Of the microvessels, the diameters are irregular and the running is asymmetric. The two-dot chain line demarcates the carcinomatous and noncarcinomatous areas.

(d) Ultra-fine granular pattern (u-FGP) characterized by the presence of microvessels that are perceived as minute brownish points and surface structures composed of ultra-fine granules with slightly uneven sizes and distributions in the depressed area.

Microvascular disruption is absent in all of the four NBI-ME patterns.

**Table 3:** Patient characteristics.

Type (number of lesions/patients)	Simple type (*n* = 30/25)	Mixed type (*n* = 15/14)	*p* value
Age (years range)	73.5 ± 7.5 (50–84)	73.4 ± 10.4 (57–93)	0.776
Sex, male/female	16/9	11/3	0.336
Size (mean ± SD mm)	10.4 ± 6.0	21.1 ± 17.1	**0.038**
Macroscopic type			
0-IIa/0-IIb/0-IIc	22/2/6	8/4/3	0.090
Location			
Lower/middle/upper	9/17/4	4/8/3	0.846
Color on WLE			
Whitish/isochromatic/reddish	20/3/7	7/3/5	0.414
Endoscopic atrophic pattern^*∗*^			
C-1, C-2/C-3, O-1/O-2, O-3	0/3/22	0/1/14	0.485
NBI-ME type			
RMP/LIPPS/MLP/u-FGP	20/3/4/3	—	—
Recognizability of lateral border on WLE (on NBI-ME)			
Clear/unclear	22/8 (28^a^/2)	9/6 (12/3)	^a^0.004
Mucin phenotype			
Gastric/gastrointestinal/intestinal	2^b^/11/17^b^	7^b^/4/4	^b^0.007
*Helicobacter pylori*			
Positive/negative^*∗∗*^	14/11	7/7	0.719

SD, standard deviation; 0-IIa, superficial elevated type; 0-IIb, superficial flat type; 0-IIc, superficial depressed type; WLE, white light endoscopy; *∗*, Kimura-Takemoto classification; NBI-ME, magnifying endoscopy with narrow-band imaging; RMP, regular mesh pattern; LIPPS, loops within irregular polygonal and papillary structures; MLP, maze-like pattern; u-FGP, ultra-fine granular pattern; *∗∗*, including the patients who had severe atrophic gastritis and were considered after natural eradication of *Helicobacter pylori*.

^a^The recognizability of lateral border on NBI-ME was significantly higher than that on WLE in the S-LG-tub1 group.

^b^Immunohistochemistry revealed that the frequency of lesions of the gastric (G) phenotype in the M-LG-tub1 group (53.3%) and the intestinal (I) phenotype in the S-LG-tub1 group (56.7%) were significantly higher and the gastric (G) phenotype in the S-LG-tub1 group (6.7%) was significantly lower than that of other phenotypes in both groups (*p* = 0.007).

**Table 4:** NBI-ME pattern types and interobserver agreement for diagnosing S-LG-tub1.

	Observer 2					
		RMP	LIPPS	MLP	u-FGP	*k*
Observer 1	RMP	16	0	4	0	0.859
	LIPPS	0	3	0	0	
	MLP	0	0	4	0	
	u-FGP	0	0	0	3	

NBI-ME, magnifying endoscopy with narrow-wband imaging; S-LG-tub1, simple-type, low-grade, well-differentiated tubular adenocarcinoma; RMP, regular mesh pattern; LIPPS, loops within irregular polygonal and papillary structures; MLP, maze-like pattern; u-FGP, ultra-fine granular pattern.

**Table 5:** The sensitivity, specificity, accuracy, PPV, NPV, and intra- and interobserver agreement using the “limited-to-four-pattern sign” observed on NBI-ME for the differential diagnosis between S-LG-tub1 and M-LG-tub1.

Measure	Observer I-1 (first observation)			Observer I-2 (second observation)			Observer II	
	%	(95% CI)		%	(95% CI)		%	(95% CI)
Sensitivity	90.9	(84.2–90.9)		93.8	(87.1–93.8)		87.9	(80.1–90.3)
Specificity	100.0	(81.5–100.0)		100.0	(83.5–100.0)		91.7	(70.4–98.4)
Accuracy	93.3	(83.5–93.3)		95.6	(86.0–95.6)		88.9	(77.6–92.5)
PPV	100.0	(92.6–100.0)		100.0	(92.9–100.0)		96.7	(88.2–99.4)
NPV	80.0	(65.2–80.0)		93.3	(83.5–93.3)		73.3	(56.3–78.8)
*k* value								
Interobserver						0.737		
Intraobserver			0.842					

PPV, positive predictive value; NPV, negative predictive value; NBI-ME, magnifying endoscopy with narrow-band imaging; M, mixed-type; S, simple-type; LG-tub1, low-grade, well-differentiated tubular adenocarcinoma. The “limited-to-four-pattern (LFP) sign-positive” means that the NBI-ME findings exhibiting more than one of the four pattern types (regular mesh pattern, (RMP), loops within irregular polygonal and papillary structures (LIPPS), maze-like pattern (MLP), and ultra-fine granular pattern (u-FGP)) mentioned in this research can be observed in a single lesion without other NBI-ME patterns that could not be classified into any of the four aforementioned types, whereas the “LFP sign-negative” on NBI-ME means that the NBI-ME findings exhibiting more than one of the four aforementioned pattern types can be observed in a single lesion with other patterns.
